# Fast and furious: Early differences in growth rate drive short‐term plant dominance and exclusion under eutrophication

**DOI:** 10.1002/ece3.6673

**Published:** 2020-09-09

**Authors:** Pengfei Zhang, Mariet M. Hefting, Merel B. Soons, George A. Kowalchuk, Mark Rees, Andy Hector, Lindsay A. Turnbull, Xiaolong Zhou, Zhi Guo, Chengjing Chu, Guozhen Du, Yann Hautier

**Affiliations:** ^1^ Ecology and Biodiversity Group Department of Biology Utrecht University Utrecht The Netherlands; ^2^ State Key Laboratory of Grassland and Agro‐Ecosystems School of Life Sciences Lanzhou University Lanzhou China; ^3^ Institute of Eco‐Environmental Forensics of Shandong University Jinan China; ^4^ Ministry of Justice Hub for Research & Practice in Eco‐Environmental Forensics Qingdao China; ^5^ Department of Animal and Plant Sciences Western Bank University of Sheffield Sheffield UK; ^6^ Department of Plant Sciences University of Oxford Oxford UK; ^7^ Institute of Arid Ecology and Environment Xinjiang University Urumqi China; ^8^ Department of Ecology State Key Laboratory of Biocontrol and School of Life Sciences Sun Yat‐sen University Guangzhou China

**Keywords:** competitive dominance, diversity loss, early growing season, Eutrophication, exclusion, growth rate, I* theory, R* theory, short‐term competition

## Abstract

1. The reduction of plant diversity following eutrophication threatens many ecosystems worldwide. Yet, the mechanisms by which species are lost following nutrient enrichment are still not completely understood, nor are the details of when such mechanisms act during the growing season, which hampers understanding and the development of mitigation strategies.
2. Using a common garden competition experiment, we found that early‐season differences in growth rates among five perennial grass species measured in monoculture predicted short‐term competitive dominance in pairwise combinations and that the proportion of variance explained was particularly greater under a fertilization treatment.
3. We also examined the role of early‐season growth rate in determining the outcome of competition along an experimental nutrient gradient in an alpine meadow. Early differences in growth rate between species predicted short‐term competitive dominance under both ambient and fertilized conditions and competitive exclusion under fertilized conditions.
4. The results of these two studies suggest that plant species growing faster during the early stage of the growing season gain a competitive advantage over species that initially grow more slowly, and that this advantage is magnified under fertilization. This finding is consistent with the theory of asymmetric competition for light in which fast‐growing species can intercept incident light and hence outcompete and exclude slower‐growing (and hence shorter) species. We predict that the current chronic nutrient inputs into many terrestrial ecosystems worldwide will reduce plant diversity and maintain a low biodiversity state by continuously favoring fast‐growing species. Biodiversity management strategies should focus on controlling nutrient inputs and reducing the growth of fast‐growing species early in the season.

## Graphical abstract

The mechanisms underlying species loss following nutrient enrichment are still not completely understood, as are the details of exactly when such mechanisms act during the growing season, which hampers the development of mitigation strategies. Using a common garden competition experiment with five perennial grass species and a fertilization treatment, we found that early‐season differences in growth rates measured in monocultures predicted short‐term competitive dominance in pairwise combinations and that this effect was magnified under fertilization. We then examined the role of early‐season growth rate in determining the outcome of competition along a manipulated nutrient gradient in an alpine meadow. Early differences in growth rate between species predicted short‐term competitive dominance under both ambient and fertilized conditions and competitive exclusion under fertilized conditions. Our results suggest that plant species growing faster during the early stage of the growing season gain a competitive advantage over species that initially grow more slowly, and that this advantage is magnified under fertilization. This finding is consistent with the theory that fast‐growing species can intercept incident light and hence outcompete and exclude slower‐growing species. We therefore predict that the current chronic nutrient inputs into many terrestrial ecosystems worldwide will continue to reduce plant diversity.

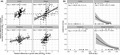

## 1 INTRODUCTION

Anthropogenic inputs of nutrients, including nitrogen (N) and phosphorus (P), into the biosphere have greatly increased in recent decades and continue to rise (Sinha, Michalak, & Balaji, 2017). This environmental eutrophication represents a major threat to biodiversity in many terrestrial, freshwater, and marine ecosystems worldwide, as it is usually associated with biodiversity loss (Borer et al., 2014; Ren et al., 2017). In grasslands, nutrient enrichment, both deliberate (agricultural fertilization) and unintentional (atmospheric deposition), has been shown to have profound impacts on ecosystems (Erisman, Sutton, Galloway, Klimont, & Winiwarter, 2008). Nutrient input usually increases primary productivity and reduces plant diversity and community stability (Midolo et al., 2018; Soons et al., 2017). This loss of plant diversity can then impact the functioning of ecosystems and their associated ecosystem services (Hautier, Isbell, et al., 2018; Hautier et al., 2014, 2015; Hector et al., 2010; Isbell et al., 2015). However, we do not have a complete understanding of the mechanisms by which nutrient inputs lead to the loss of plant diversity (Harpole et al., 2017) or the timing during the growing season when these mechanisms are most important.

In unproductive grasslands, where soil resources are strongly limiting, diversity is often high. However, resource competition theory predicts dominance by the single species that can deplete soil resources to the lowest level (i.e., the lowest value of R*) (Tilman, 1980, 1982). We must therefore assume that unproductive grasslands are either limited by more than one belowground resource (Fay et al., 2015; Hutchinson, 1957), or that additional mechanisms operate to promote the coexistence of multiple species, such as negative soil feedbacks (Petermann, Fergus, Turnbull, & Schmid, 2008). Coexistence might be made easier in such systems because competition for belowground resources is often assumed to be size‐symmetric (Hautier, Vojtech, & Hector, 2018; Vojtech, Turnbull, & Hector, 2007), thus leading to relatively small fitness differences between species, which can be offset by weak niche differentiation (Chesson, 2000).

Under productive conditions, when nutrient limitation is alleviated and light becomes the limiting resource, resource competition theory again predicts competitive dominance, this time by the species that is able to intercept light and reduce it to the lowest level (i.e., the lowest value of I*) (Dybzinski & Tilman, 2007; Vojtech et al., 2007). Because light is a directionally supplied resource, tall species can intercept and pre‐empt light, making it unavailable to low‐growing species. Competition for light is likely to be highly size‐asymmetric and might therefore lead to very large fitness differences and hence the exclusion of smaller, slow‐growing species (Borer et al., 2014; DeMalach, Zaady, & Kadmon, 2017; Hautier, Niklaus, & Hector, 2009).

While measurements of mechanistic plant competition (i.e., shoot or root) are extremely difficult, relative growth rate (RGR), the rate of accumulation of new dry mass per unit of existing dry mass in a given time interval, is relatively easy to measure, and many plant species show striking differences in their relative growth rate, even when grown under similar environmental conditions (Grime & Hunt, 1975). High RGR might confer a strong competitive advantage under productive conditions, because it enables a species to quickly capture light and deny it to competitors. But under unproductive conditions, we might expect high RGR to be a weaker predictor of competitive outcomes, as other traits, reflecting niche differences, may play a greater role. The timing of growth might also be a key factor in determining competitive outcomes. For example, a species growing faster during the early stage of the growing season might reduce light availability and thus have a disproportionate competitive advantage relative to species that initially grow more slowly (Huston & Smith, 1987). As a result, differences in RGR (particularly in the early season) should result in greater differences in final biomass under more productive conditions. This is because as the importance of asymmetrical light competition increases, early size differences cumulate over time, leading to greater differences in final biomass. We thus expect the slope of the relationship between (early season) RGR and differences in final biomass to be steeper under more productive conditions. RGR can be calculated at different time points and thus be used to identify when during the growing season differences in RGR are particularly important.

We used two studies to test whether early differences in species growth rates better predict short‐term competitive dominance under productive conditions: (a) a common garden experiment where species were grown in monoculture and in pairwise and five‐species mixtures under unproductive and productive conditions and (b) an experiment in a natural grassland community that also included unproductive and productive conditions. Critically, both studies provide detailed measurements of aboveground biomass through the growing season. We compare productive conditions with unproductive conditions and focus mainly on competitive outcomes in productive conditions, where we expect competition to be primarily for light, hence species with high early‐season RGR in monoculture should dominate mixtures. We contrast the productive condition with unproductive condition but because the outcome of competition may be slower, the comparison is limited by the short‐term nature of our study.

## 2 MATERIAL AND METHODS

### 2.1 Overview

Our two experiments both measured aboveground plant biomass at regular intervals during the growing season at a relatively uncommon level of temporal detail. From these measurements, we could calculate daily RGR per species throughout the growing season, which provides unique insight into growth rates and their temporal changes (in contrast, most studies lack a temporal dimension and measure biomass only at harvest). We used these measures of RGR to identify when, during the growing season, differences in RGR best predict competitive outcomes in mixtures, comparing the high and low productive treatments in each study. The first data set comes from a competition experiment with five European perennial grass species grown under nutrient‐limited unproductive and nutrient‐rich productive conditions in the experimental garden of the University of Zurich, Switzerland (47°23′N, 8°33′E, and 546 m height a.s.l.). Mean monthly temperature ranges from 0.2°C in January to 18.4°C in July, and mean annual temperature is 9.3°C. Annual precipitation is 1,085 mm falling regularly but more during the summer period. The second data set lacks independent monocultures but comes from a field experiment in which nitrogen and phosphorus are added alone or in combination to a flat alpine meadow at the Alpine Meadow and Wetland Ecosystems Research Station of Lanzhou University (Azi Branch Station) in the eastern Tibetan Plateau (33°40′N, 101°51′E, altitude 3,500 m a.s.l.), Gansu, China. Mean monthly temperature ranges from −10°C in January to 11.7°C in July, and mean annual temperature is 1.2°C. Annual precipitation is 620 mm and falls mainly during the short, cool summer. The growing season typically starts around April 15 (~day 106) in Zürich and around May 15 (~day 136) in Gansu.

### 2.2 Common garden experiment

#### 2.2.1 Experimental design

The common garden experiment has been described at greater length elsewhere (Hautier, Vojtech, et al., 2018; Vojtech, Loreau, Yachi, Spehn, & Hector, 2008; Vojtech et al., 2007). Briefly, we established monocultures (*n* = 5), all pairwise mixtures (*n* = 10), and the full five‐species mixtures (*n* = 1) of five perennial grass species (Poaceae): *Alopecurus pratensis* L. (*Al*), *Anthoxanthum odoratum* L. (*An*), *Arrhenatherum elatius* (L.) P. Beauv. ex J. Presl & C. Presl (*Ar*), *Festuca rubra* ssp. *commutata* Gaud. (=*Festuca nigrescens* Lam.) (*F*), *Holcus lanatus* L. (*H*) (Lauber & Wagner, 2001). Each species combination was replicated five times for a total of 80 plots. Species were sown from seeds at a total target density of 1,000 seeds/m^2^ per plot divided equally among the species of each mixture (corrected based on the results of prior germination trials). Plants were established in 1 m^2^ plots on highly fertile soil (Garden humus, Ricoter). The experiment ran from April 2004 to June 2008. Plots were watered daily and regularly weeded throughout the duration of the experiment. During 2005 and 2006, plants were regularly fertilized with an NPK fertilizer corresponding to 15 g m^−2^ year^−1^ of nitrogen to create highly productive conditions. In 2007, we divided the plots into four subplots of 50 × 50 cm (Figure S1). In half of these subplots, we maintained the initial highly productive conditions by continuously adding the NPK fertilizer. In the other half of the subplots, we reduced soil fertility by a combination of the cessation of fertilization and the addition of sucrose (in five applications of 500 g m^−2^ year^−1^ during 2007 and two applications of 625 g/m^2^ in 2008). Addition of a carbon source limits nutrient availability to plants and reduces productivity due to the immobilization of nitrogen by soil micro‐organisms (Killham, 1994) and increases competition between micro‐organisms and plants for nitrate and ammonium (Bardgett, Streeter, & Bol, 2003; Schmidt, Michelsen, & Jonasson, 1997). Additionally, we crossed the productivity treatments with regular cutting of the canopy structure to create disturbed conditions (Hautier, Vojtech, et al., 2018). Calculating daily RGR per species throughout the growing season for the plots that were disturbed was not possible because of the limited number of samples between each cutting event. Here, we therefore analyze only the undisturbed productive and unproductive conditions.

#### 2.2.2 Data collection

On 10th–20th June 2008 (Days 161–171), after 2 years of treatment, aboveground plant biomass in the inner 30 × 30 cm of each subplot was harvested at soil level, sorted to species, dried at 80°C, and weighed (hereafter biomass at harvest). To estimate daily RGR of each species in monoculture, aboveground plant biomass was harvested at soil level within 10 × 10 cm quadrats in the outer 10 cm surrounding the inner 30 × 30 cm of each subplot during sequential harvests on days 53, 67, 88, 109, 116, 123, 130, 145, 152, 162, and 171 in the year 2008. Day 171 was the peak standing biomass. Each time different randomly chosen quadrats were measured. One 10 × 10 cm quadrat was sampled from each subplot on each of the days listed. Each time a different randomly chosen quadrat was clipped in monoculture (Figure S1). Harvested biomass samples were dried at 80°C and weighed. Soil cores were collected regularly during the growth season in 2008 and analyzed for nitrate and ammonium concentrations (Labor für Boden‐und Umweltanalytik). One soil core was collected randomly from one replicate of each monoculture, one replicate of each two‐species mixtures and three replicates of the five‐species mixtures. We measured plot level light interception ability in monoculture for each species and each nutrient treatment before the harvest in end‐April 2008 as the percentage of transmitted photosynthetically active radiation (PAR) reaching the soil surface in the inner 30 × 30 cm of each subplot.

### 2.3 Field experiment

#### 2.3.1 Experimental design

The field experiment was set up in April 2011 and has been described elsewhere (Zhang, Zhou, Li, Guo, & Du, 2015; Zhou et al., 2017; Zhou, Liu, Zhang, Zhi, & Du, 2018). Large herbivores were excluded between March and October by fencing the experimental area. A homogeneous area of meadow covering 230 × 100 m was divided into four parts that were given N, P, their combination or neither. Six plots, each 10 × 20 m, were established within each nutrient area. Fertilization treatments consisted of a factorial combination of N and P addition applied annually to fertilized plots in each of three blocks: N, P, and NP. Nitrogen was supplied at a rate of 15 g N m^−2^ year^−1^, phosphorus at a rate of 8 g P m^−2^ year^−1^, and nitrogen and phosphorus at a rate of 10 g N m^−2^ year^−1^ and 8 g P m^−2^ year^−1^. While we acknowledge that plots within each nutrient area are not independent, previous studies have shown that there were no significant differences among them in term of plant species diversity, community biomass, and community composition at the start of the experiment (Zhou et al., 2018). N was applied as ammonium nitrate (NH_4_NO_3_) and P as monocalcium phosphate (Ca(H_2_PO_4_)_2_) annually at the end of May. Each plot was subsequently divided into two subplots; one was used to measure aboveground individual biomass through time for twenty common species (Table S1), and the other was used to measure aboveground plant biomass and species composition in early August (see below in Data collection).

#### 2.3.2 Data collection

In 2013, after 3 years of nutrient addition, in the subplots dedicated to measuring aboveground individual biomass through time, we sampled twenty common species accounting for 85 ± 10% of aboveground biomass (16, 2, 1, and 1 species from Forbs, Grasses, Sedges, and Legumes respectively; Table S1). For each species, we randomly selected, dried at 80°C and weighed 12 individuals on days 146, 157, 167, 177, 197, 207, 238, and 254 in the year of 2013. We stopped sampling species once they were in full flower, resulting in a lower number of species sampled after day 177. In the subplots dedicated to measuring aboveground plant biomass and species composition at peak biomass, the vegetation was clipped in mid‐August 2013 at soil level in one randomly selected 0.5 × 0.5 m quadrat, sorted to species, dried at 80°C and weighed.

To evaluate the relative importance of RGR versus other traits likely to influence competitive ability, in 2016, we measured three functional traits for the twenty common species in each treatment: final height, specific leaf area (SLA), and leaf dry mass content (LDMC). These traits generally define species resource utilization strategies in terrestrial ecosystems (Grime, 2006). For each species, following the flowering phase, we randomly sampled nine fully developed and undamaged leaves. We weighed and scanned fresh leaves to measure leaf area using ImageJ software (Schneider, Rasband, & Eliceiri, 2012). We then dried material at 70°C for 48 hr and weighed the dried leaves. We calculated SLA as the ratio of leaf area to dry leaf mass and LDMC as the ratio of dry leaf mass to fresh leaf mass. We also randomly selected 30 flowering individuals of each species to measure the species’ final height in each treatment.

### 2.4 Statistical analyses

All analyses were done using R 3.5.1 (R Development Core Team, 2018).

#### 2.4.1 Common garden experiment

In the second year of our common garden experiment, we tested the effect of nutrient treatments on mineral nitrogen available to plants, biomass production, and understory light availability by performing ANOVA‐type generalized linear models (McCullagh & Nelder, 1989) since our response includes variables with normal and non‐normal error distributions. Data that were analyzed using normal error distribution included nitrogen available to plants and biomass production. Data with non‐normal error distribution included the percentage of understory light availability, which was analyzed with a quasibinomial error distribution to control for overdispersion.

To model plant growth, we fitted a four‐parameter logistic curve to species biomass data through time (Paine et al., 2012; Pinheiro & Bates, 2000) using a nonlinear mixed‐effects regression model with the nlme function (Pinheiro & Bates, 2000). This model best‐fitted plant growth through the season which initially increases, stabilizes and then decreases over time but not necessarily in a symmetric way. Species, nutrient treatments, and their interaction were treated as fixed effects, and the four parameters of the logistic growth model ($K,\;\text{xmid},\;M_{0}$, and r) were treated as random effects allowing them to vary between species and nutrient treatments. To improve homoscedasticity of the residuals, aboveground biomass was natural log transformed before analyses giving:

(3) $$\log\left(M_{t}\right)=M_{0}+\frac{\left(K‐M_{0}\right)}{1+\exp((\text{xmid}‐t)/r)}$$

where t is time in days of the year, $M_{t}$ is aboveground plant biomass at time t; $M_{0}$ is the asymptotic mass as t→‐∞; K is the asymptotic mass as t→∞; xmid is the mass at the inflection point, the time at which RGR is maximized and r is a scale parameter.

RGR is given by $d(\log\left(M_{t}\right))/dt$, thus we estimated daily RGR during the growing season for each species as:

(2) $${\text{RGR}}_{t}=\frac{\left(K‐M_{0}\right)\exp\left(\left(\text{xmid}‐t\right)/r\right)}{r{\left(1+\exp\left(\left(\text{xmid}‐t\right)/r\right)\right)}^{2}}$$

Thus, for each species in each nutrient treatment combination, one value for RGR*_t_* was generated for each day between the first and last day of the sequential harvests, yielding 119 values of RGR*_t_* between day 53 and 171. Note that such measure of RGR is size‐standardized and has been found to be a better predictor of the short‐term outcome of competition in crowded environments than other common measure of RGR (Fakheran et al., 2010).

To assess whether early differences in growth rate between species in monocultures predict short‐term competitive dominance at harvest in pairwise and in five‐species mixtures under both productive and unproductive conditions, we related the relative differences in species biomass of the harvest of June 2008 for each pairwise mixture and for each combination of pairs in the five‐species mixtures to the daily relative differences in growth rates of the respective species and nutrient treatment combination in monoculture, thus generating 119 regressions for each of the pairwise and five‐species mixtures, one for each day between day 53 and 171.

Relative difference in abundance at harvest (day 171) in mixtures $\left(\Delta B_{\mathrm{ij}}\right)$ between species *i* and *j* was calculated as the natural log ratio of differences in biomass for each of the pairwise and five‐species mixtures as:

(3) $$\left(\Delta B_{\mathrm{ij}}\right)=\text{Ln}\left(\frac{B_{i}}{B_{j}}\right)$$

We used the natural logarithm of the response ratio to facilitate interpretation: Log response ratio is normally distributed around zero. Thus, a positive value of relative difference in abundance means that the biomass of species *i* at harvest is higher than that of species *j* when grown together, that is, species *i* has a greater relative abundance when grown with species* j*, and vice versa. Ten values of relative difference in abundance ($\Delta B_{\mathrm{ij}}$) were calculated for each of the pairwise and five‐species mixtures, one for each of the ten combination of pairs of species.

Daily relative differences in growth rates $\left(\Delta {\text{RGR}}_{\mathrm{tij}}\right)$ between species *i* and *j* were calculated for each day between day 53 and 171 as the natural log ratio of difference in RGR in monoculture as:

(4) $$\left(\Delta {\text{RGR}}_{\mathrm{tij}}\right)=\text{Ln}\left(\frac{{\text{RGR}}_{\mathrm{ti}}}{{\text{RGR}}_{\mathrm{tj}}}\right)$$

A positive value of daily relative differences in growth rates means that the relative growth rate in monoculture at time *t* of species *i* is higher than that of species* j*, that is, species *i* grow relatively faster than species *j* at a given day in the year, and vice versa. For each of the ten species pairs, daily relative differences in growth rates ($\Delta {\text{RGR}}_{\mathrm{tij}}$) were calculated for each day between day 53 and 171 for the pairwise and five‐species mixtures.

We assessed the relationship between the relative differences in species abundance in mixture (pairwise and full five species) and daily relative differences in growth rates using generalized linear models with a normal error distribution. The relative differences in abundance in mixture were the response variable and relative differences in growth rates, nutrient treatments, and their interaction were the explanatory variables. A positive relationship would indicate that species with a higher RGR at time *t* have greater competitive ability and aboveground biomass at harvest. For each regression, we extracted the slope and 95% CI as well as the percentage of variance explained (*R* ^2^ value).

#### 2.4.2 Field experiment

In the third year of our field experiment, we tested the individual and interactive effects of nitrogen and phosphorus addition on biomass production and plant species richness by performing ANOVA. We used generalized linear models with normal error distributions for both variables as each variable followed a normal distribution.

To quantify plant growth, we fitted a four‐parameter logistic growth model to species biomass data through time using a nonlinear mixed‐effects regression model with Equations 1 and 2 yielding 109 values of RGR*_t_* between day 146 and 254.

To assess whether early differences in growth rate between species predict short‐term competitive dominance in real‐world ecosystem, we related the relative differences in abundance at harvest and daily relative differences in growth rates between day 146 and 254 for each combination of pairs of species in a treatment combination using Equations 3 and 4, respectively, thus generating 109 regressions, one for each day between day 146 and 254 during the growing season in 2013. Because of the lack of a randomized blocked design, we fitted separate models for each treatment and compared the estimates informally.

Calculations of RGR*_t_* in the field study are based on species growing in mixtures (in the common garden experiment these were based on species growing in monocultures). In this case, *K*, the asymptotic mass in mixture, is a direct measure of competitive ability. Hence, we would expect competitive dominants to have high *K* values and therefore high RGR*_t_*. We thus run a simple additional analysis in which we calculated RGR as $\log\left(\frac{B1}{B0}\right)/t$ where *B*0 and *B*1 are the first and second measurements of biomass and *t* the time between. We then related relative difference in abundance at harvest to relative differences in growth rates for each combination of pairs of species in a treatment combination using Equations 3 and 4, respectively. Such analysis does not include the *K* parameter and thus does not include the dependence between the asymptotic size in mixtures and competitive ability. Results did not differ qualitatively between the two analyses, indicating that results based on the RGR*_t_* measured with species growing in mixtures is not an artifact of the fitted models.

We assessed the relationship between the relative differences in abundance in mixture and daily relative differences in growth rates using generalized linear models with a normal error distribution. The relative abundance in mixture was the response variable and relative differences in growth rates, nutrient treatments, and their interaction were the explanatory variables. A positive relationship would indicate that species with a higher RGR at time *t* have greater competitive ability and aboveground biomass at harvest.

We assessed the relative contribution of each trait (height, SLA, and LDMC) and RGR*_t_* to predict the abundance in mixtures by using a multivariate model to calculate the percentage of variance explained (percentage of *R* ^2^). Each trait and RGR*_t_* were fitted as main effect explanatory variables and abundance in mixtures as response variable. The model was fitted for the day *t* at which the percentage of variation explained by the regression (*R* ^2^) was maximum for each nutrient addition combination (see Figure 4). As for the approach described above, we calculated the natural logarithm of the response ratio for each variable before fitting the multivariate model. RGR*_t_* was weakly positively correlated with height (*r* = .15 (95% CIs = 0.06–0.25), *df* = 404) and LDMC (*r* = .26 (95% CIs = 0.17–0.35), *df* = 404) and weakly negatively correlated with SLA (*r* = −.31 (95% CIs = −0.40 to −0.22), *df* = 404).

We assessed whether early differences in species growth rate predict short‐term competitive exclusion in the nutrient addition treatment using generalized linear models with a quasibinomial error distribution. A species was considered lost when it was present in a plot in 2011 and absent from that plot in 2013, leading to a 1 if the species was present in 2011 and 2013 and a 0 if it was present in 2011 but absent in 2013. We related the likelihood of a species to be lost after 3 years of nutrient addition to daily RGR values for that species, thus generating 109 regressions, one for each day between day 146 and 254 during the growing season in 2013. The likelihood of a species being lost was the response variable, and RGR values and nutrient treatments were the explanatory variables. A negative relationship would indicate that species with a higher RGR at time *t* have greater competitive ability and exclude species with lower RGR. For each regression, we extracted the slope and 95% CI as well as the percentage of variance explained (*R* ^2^ value).

## 3 RESULTS

### 3.1 RGR predicts short‐term competitive dominance in a common garden experiment

Our nutrient addition treatment successfully created productive condition with high nutrient and low light availability, while our sucrose addition treatment successfully created relatively unproductive condition with limited nutrient and high light availability. Sucrose addition reduced the amount of mineral nitrogen available to plants, in the second year of our common garden experiment, from an average of 2.3 ± 0.3 g N m^−2^ following nutrient addition to 0.9 ± 0.3 g N m^−2^ following sucrose addition. It also reduced biomass production in monocultures from 745 ± 39 g/m^2^ (mean ± *SEM*) following nutrient addition to 274 ± 25 g/m^2^ following sucrose addition (*F*_1,48_ = 102.34, *p* < .001) and increased understory light availability measured just before the harvest from 13 ± 3% following nutrient addition to 65 ± 5% following sucrose addition (*F*_1,48_ = 54.25, *p* < .001) (Table S2).

**FIGURE 1:**
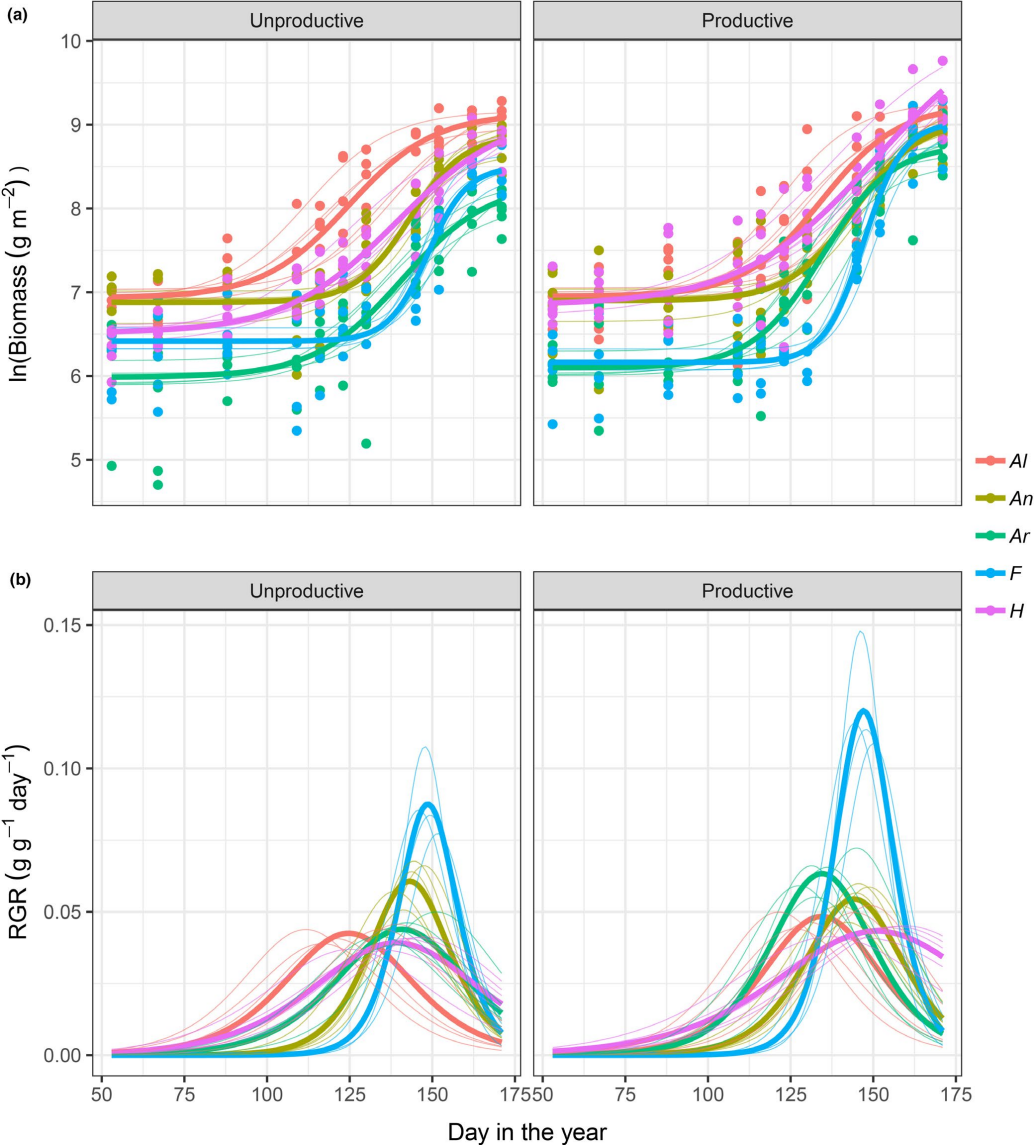
Common garden. Fitted curves predicted from a four‐parameter logistic model for (a) biomass (log transformed) and (b) relative growth rate (RGR) over time for five perennial grass species from the monoculture plots grown under unproductive (left panels) and productive (right panels) conditions. Thicker lines are average fitted curves across the five replicated plots for each species while thinner are fitted curves for each species and each replicated plot. *Al* = *Alopecurus pratensis*, *An* = *Anthoxanthum odoratum*, *Ar* = *Arrhenatherum elatius*, *F* = *Festuca rubra,* and *H* = *Holcus lanatus*

After 2 years of treatment, the four parameters of the logistic growth curves used to calculate daily RGR of five perennial grass species growing in monoculture varied across species and nutrient treatments (Figure 1a; Table S3). As a result, the rankings for species’ growth rates changed with both the growing season and nutrient treatment (Figure 1b). For example, relatively high RGR early in the season was observed for *H. lanatus* under productive condition, while *A. pratensis* had the highest early RGR under unproductive condition.

**FIGURE 2:**
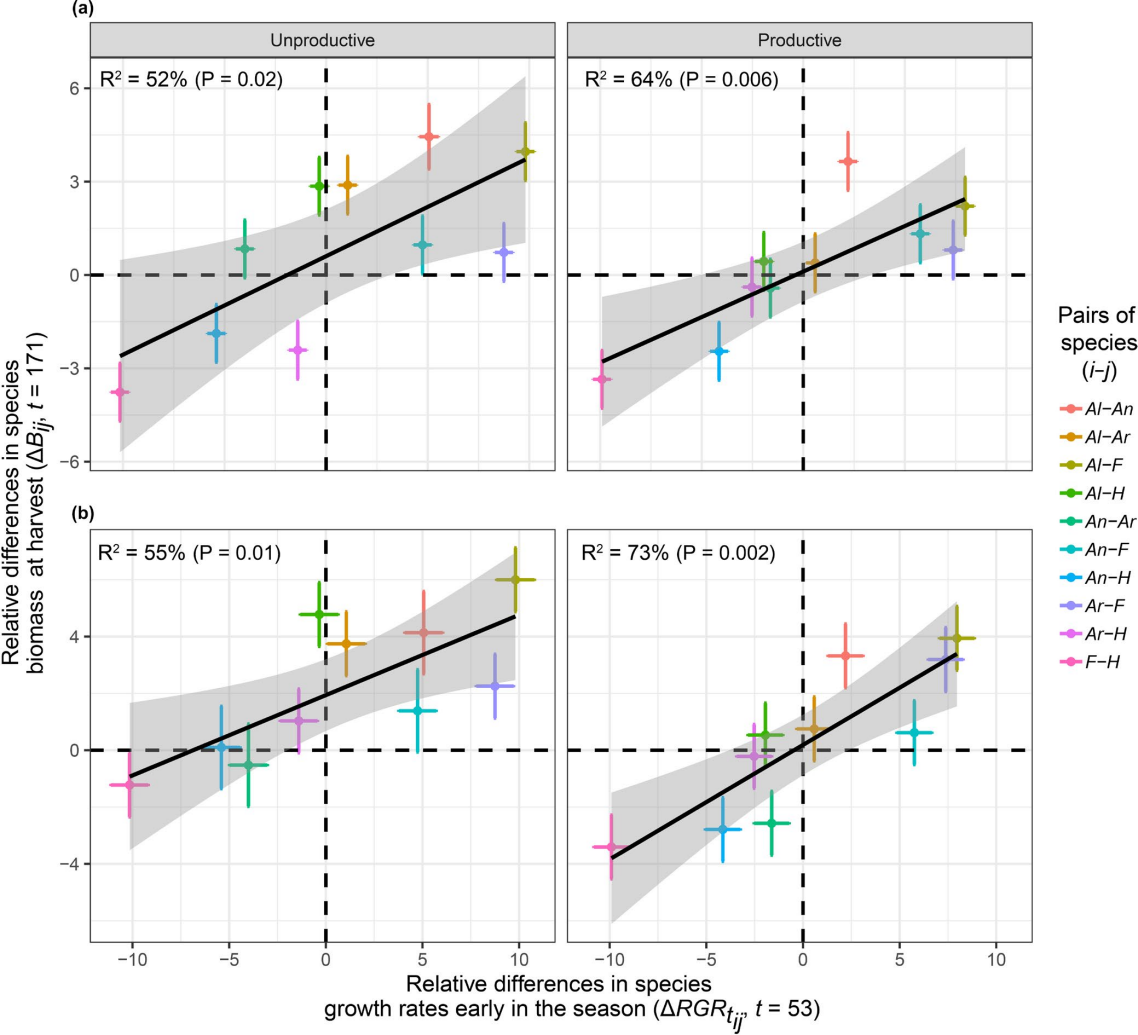
Common garden. Relationships of the biomass ratio at harvest date (day 171) of each pairwise mixture (*B_ij_*) with the daily RGR ratio of the respective species in monoculture (RGR*_tij_*) in (a) pairwise combinations and (b) five‐species mixtures under both unproductive (left panels) and productive (right panels) conditions. Black points are the slope of the relationships for each day between day 53 and 171 in 2008. The shaded green area represents the 95% confidence intervals around the slopes. Red points are the percentage of variance explained by each relationship. The vertical dashed line represents the time point at which the slope of the relationship switched between positive and negative

**FIGURE 3:**
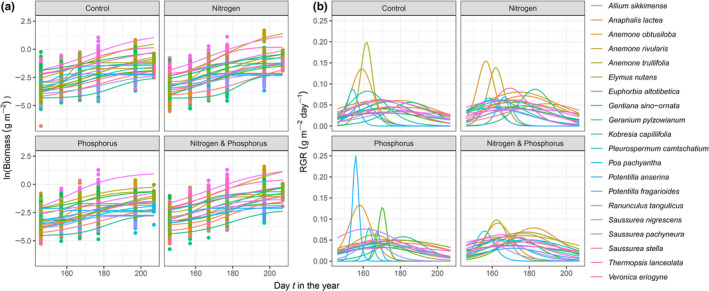
Common garden. Early‐season relative differences in species growth rates in monoculture (${\text{RGR}}_{\mathrm{tij}},\;t=53$) predict relative differences in species biomass (*B_ij_*) at harvest date (t=171) in (a) ten pairwise mixtures of five species and (b) ten combination of pairs of species within five‐species mixtures under unproductive (left panels) and productive (right panels) conditions. Relative differences were calculated as the natural logarithm of the ratio between pairs of species in a treatment combination. *Al* = *Alopecurus pratensis*, *An* = *Anthoxanthum odoratum*, *Ar* = *Arrhenatherum elatius*, *F* = *Festuca rubra*, and *H* = *Holcus lanatus*. Means ± 95% confidence intervals are shown. The gray region indicates the 95% confidence interval around the regression

We found that early‐season relative differences in species growth rates in monoculture were positively associated with relative differences in species biomass at harvest (day 171) in pairwise (Figure 2a, Figure 3a) and five‐species mixtures (Figure 2b, Figure 3b) under both productive and unproductive conditions and remarkably similar in pairwise combinations and five‐species mixtures under nutrient‐rich conditions. The slope of the relationship was steeper under unproductive condition in pairwise mixtures compared to productive conditions (Figure 2a), but steeper under productive condition in five‐species mixtures compared to unproductive conditions (Figure 2b). The percentage of variance explained was maximum between day *t* = 53 and *t* = 112 (Figure 2). This positive association was observed up to day *t* = 133 and was not significantly different between the productive and unproductive conditions for the pairwise mixtures (Table S4), but was significantly weaker under unproductive compared with productive conditions for the five‐species mixtures (Table S5). Relative differences in species’ growth rates became smaller as the season progressed until they became negatively associated with differences in species biomass (from day *t* = 135) (Figure 2). The percentage of variance in species biomass differences at harvest explained by relative differences in species’ growth rates during the early stage of the growing season was approximately 60% under the productive condition and 50% under the productive condition for the pairwise mixtures (Figure 2a) and approximately 75% under the productive condition and 55% under the unproductive condition for the five‐species mixtures (Figure 2b).

### 3.2 RGR predicts short‐term competitive dominance and exclusion in a field experiment

Our nitrogen and combined nitrogen and phosphorus addition treatments created productive conditions and reduced plant diversity while phosphorus addition alone did not significantly affect either productivity or diversity. In the third year of our field experiment, there was a marginally nonsignificant interaction between nitrogen and phosphorus addition on biomass production (*F*_1,20_ = 3.8, *p* = .065) and plant species richness (*F*_1,20_ = 3.7, *p* = .069) (Table S6). Nitrogen addition increased biomass production from an average of 101 ± 11 g 0.25 m^−2^ (mean ± *SEM*) in the control plots to 140 ± 11 g 0.25 m^−2^ and decreased species richness from 36 ± 2 species 0.25 m^−2^ to 22 ± 2 species 0.25 m^−2^. In contrast, the levels of biomass production (114 ± 11 g 0.25 m^−2^) and species richness (35 ± 2 species 0.25 m^−2^) under phosphorus addition did not differ significantly from those observed in the control plots. The combination of nitrogen and phosphorus addition had a large effect on productivity, which increased to 198 ± 11 g 0.25 m^−2^, while this treatment resulted in a smaller but still significant decrease in plant species richness than observed with just nitrogen treatment leading to 28 ± 2 species 0.25 m^−2^.

**FIGURE 4:**
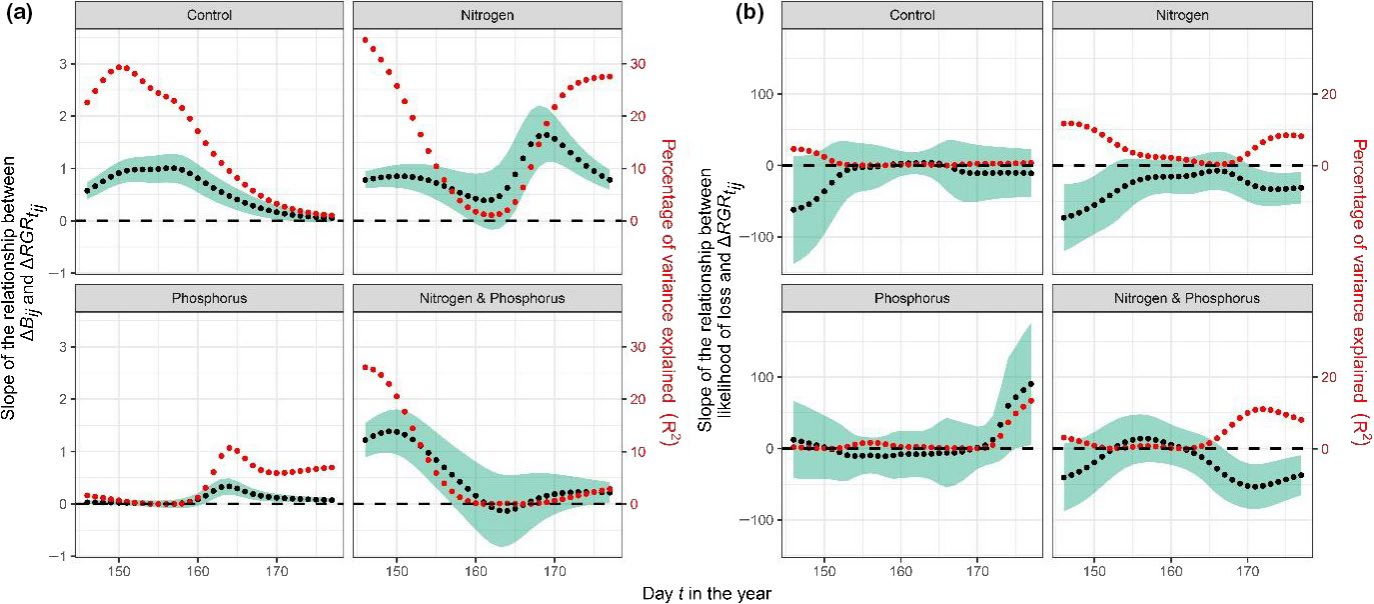
Field experiment. Relationships of (a) the biomass ratio (*B_ij_*) at harvest date (day 213–221) of each combination of pairs of species in each nutrient addition combination with the daily RGR ratio of the respective species and nutrient addition combination (${\text{RGR}}_{\mathrm{tij}}$), and (b) the likelihood of a species to be lost after 3 years of nutrient addition with daily RGR values for that species (${\text{RGR}}_{\mathrm{tij}}$). Black points are the slope of the relationships for each day between day 146 and 254 in 2013. Red points are the percentage of variance explained by each relationship. The shaded green area represents the 95% confidence intervals around the slopes

**FIGURE 5:**
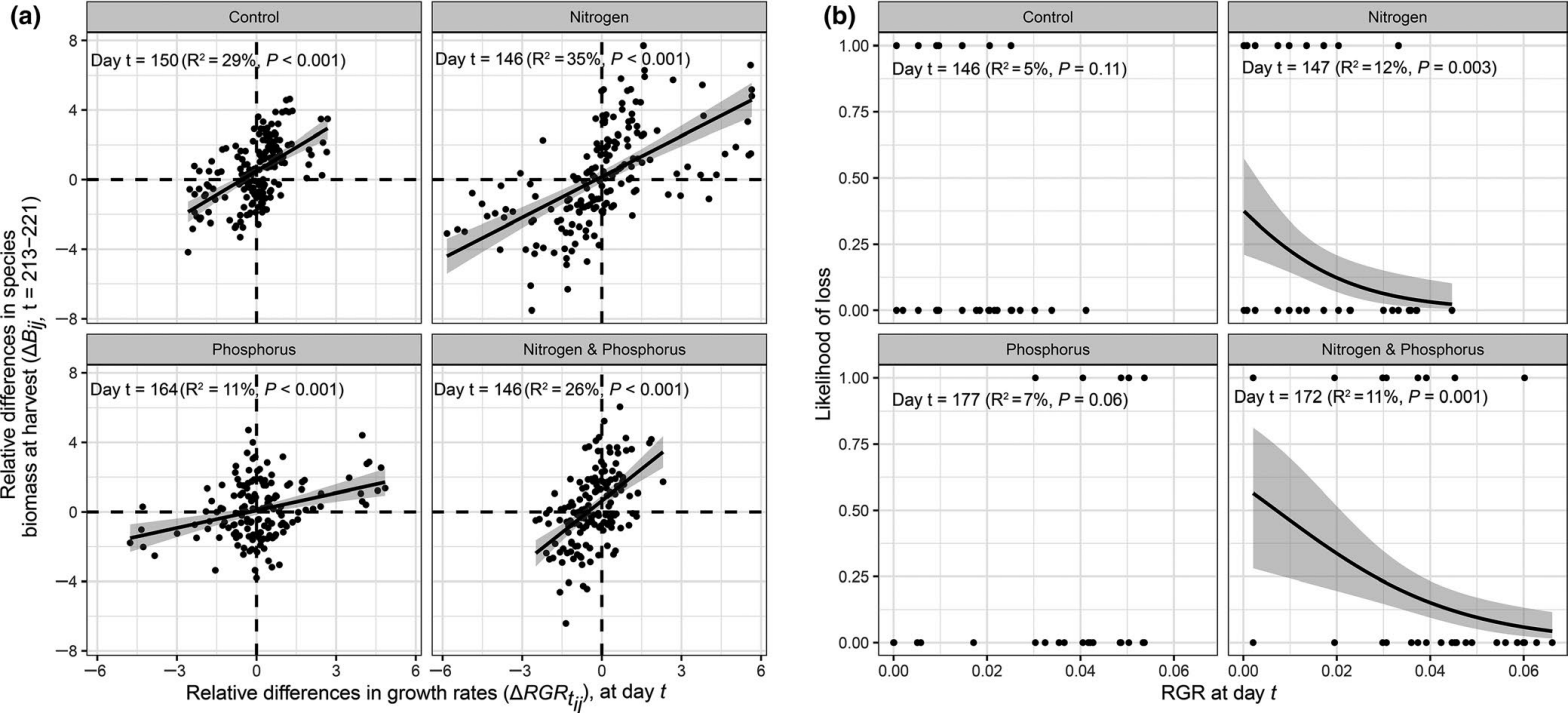
Field experiment. RGR predicts competitive dominance and exclusion. (a) Early‐season relative differences in species growth rates in a nutrient addition combination (${\text{RGR}}_{\mathrm{tij}}$) predict relative differences in species biomass in pairs of species combinations of the respecting nutrient addition combination (*B_ij_*) at harvest date (t=213‐221). B) Early‐season growth rate in a nutrient addition combination (RGR) predicts the likelihood of a species to be lost in the respecting nutrient addition combination (Likelihood of loss; a species was considered lost when it was present in a plot in 2011 and absent from that plot in 2013). Dots in (b) indicate RGR at day *t* of species that were lost (1) or not lost (0). Results are shown for the day *t* at which the percentage of variation explained by the regression (*R* ^2^) was maximum for each nutrient addition combination (see Figure 4). Within each graph (a, b) productive conditions with added *N* (right) are separated from unproductive conditions without added *N* (left). The gray region indicates the 95% confidence interval around the regression

Similar to the results of our common garden experiment, rankings of species growth rates changed with both growing season and nutrient treatments (Figure S2). We found the relative differences in species growth rates to be the strongest contributor to relative differences in species biomass compared with the other functional traits measured. Specifically, in terms of relative contribution to explained variation in species biomass differences, differences in species growth rates explained about 50% of the variation, SLA about 26%, LDMC about 14%, and height about 10%. The relationship between early‐season relative differences in species growth rates and relative differences in species biomass varied across both the growing season and nutrient treatments (Figure 4a, Figure 5a). The percentage of variance explained was maximum at day 150 in the control (*R* ^2^ = .29, *F*_1,169_ = 70.1, *p* < .001), 146 with nitrogen addition (*R* ^2^ = .35, *F*_1,169_ = 89.3, *p* < .001), 164 with phosphorus addition (*R* ^2^ = .11, *F*_1,151_ = 18.0, *p* < .001), and 146 with nitrogen and phosphorus addition (*R* ^2^ = .26, *F*_1,151_ = 53.2, *p* < .001). When significant, relationships were always positive (Figure 4a, Figure 5a). Results based on relative differences in species growth rates calculated between the two first measurements of biomass confirmed that early differences in growth rates predict competitive dominance at harvest except in the phosphorus addition treatment (Figure S3).

We found that the relationship between early‐season species growth rate values and the likelihood of loss of a species varied with both the growing season and nutrient treatments (Figure 4b, Figure 5b). The percentage of variance explained was maximum at day 146 in the control (*R* ^2^ = .05, *F*_1,118_ = 2.9, *p* = .11), 147 with nitrogen addition (*R* ^2^ = .12, *F*_1,118_ = 11.5, *p* = .003), 177 with phosphorus addition (*R* ^2^ = .07, *F*_1,112_ = 3.6, *p* = .06), and 172 with nitrogen and phosphorus addition (*R* ^2^ = .11, *F*_1,118_ = 11.6, *p* = .001). Short‐term competitive exclusion could only be predicted by early differences in species growth rate under productive conditions (nitrogen and nitrogen & phosphorus addition) and, when significant, relationships were always negative indicating that species with a higher RGR at time *t* have greater competitive ability and exclude species with lower RGR (Figure 4b, Figure 5b). Under unproductive conditions (control and phosphorus addition), short‐term competitive exclusion could not be predicted from early differences in growth rate.

## 4 DISCUSSION

Our competition experiment in a common garden shows that early‐season differences in species’ growth rates in monoculture are good predictors of short‐term differences in relative abundance in pairwise and five‐species mixtures and that differences in RGR explained a greater proportion of variance in relative abundance under productive (light‐limited) conditions. The species that grew faster early in the season (i.e., *H. lanatus* and *A. pratensis)* had the greatest competitive advantage relative to slower‐growing species (i.e., *A. odoratum*, *A. elatius,* and *F. rubra*). Relative differences in species growth rates became smaller as the growing season progressed until they eventually became negatively associated with differences in species biomass. This switch corresponds to the time at which faster‐growing species had already reached their maximum growth rate and gradually slowed down while the RGR of slow‐growing species was still rising (day *t* = 135). Early differences in species’ growth rate also governed short‐term competitive outcomes in our seminatural grassland subjected to nutrient addition, thereby extending the results of the common garden experiment to a real‐world grassland ecosystem. Together these results indicate that species growing faster during the early stage of the growing season, and thus reducing light availability during this early phase of vegetation growth, had a competitive advantage relative to species that initially grow more slowly.

We found that the proportion of variance explained was particularly greater and the slope of the relationship was particularly steeper under productive conditions in the five‐species mixtures. This suggests that additional processes that play an important role in promoting the coexistence of competitors in multispecies communities may be disrupted under more fertile and productive conditions. One possible explanation is that productive conditions could reduce the similarity of competitive abilities and thus reduce intransitive competition (Soliveres et al., 2018). This could be due to a reduction of niche dimensionality and potential for trade‐offs for belowground limiting nutrients (Harpole et al., 2016, 2017; Harpole & Tilman, 2007), or to an increase in the asymmetry of competition from nutrients to light leading to dominant species competing mainly for a single resource which makes competition less intransitive (DeMalach et al., 2017; Hautier et al., 2009).

Numerous plant traits interactively determine differences in relative growth rate and abundance in a manner predictable from trait‐based theory (McGill, Enquist, Weiher, & Westoby, 2006; Westoby & Wright, 2006). However, we found weak correlation between differences in relative growth rate and differences in height, SLA, and LDMC. Additionally, we found that early differences in relative growth rate were far better predictor of differences in abundance at harvest as compared to difference in these traits. While we measured only some of the potential functional traits dictating differences in abundance, our results support our hypothesis that species growing faster during the early stage of the growing season gain a competitive advantage over species that initially grow more slowly.

Addition of nitrogen in our seminatural grassland ecosystem increased productivity and reduced plant diversity, allowing us to further assess whether differences in species growth rate predict short‐term competitive exclusion due to nutrient addition. We found that difference in early‐season RGR predict short‐term competitive exclusion under productive conditions, but not under unproductive conditions. Under productive conditions, the species that grew faster early in the season (e.g., *Anemone trullifolia, Gentiana sino‐ornata, and Saussurea nigrescens)*, excluded initially slower‐growing species (e.g., *Potentilla anserina, Potentilla fragarioides, Euphorbia altotibetica, and Geranium pylzowianum*).

Previous studies have shown that under productive conditions, when competition is mainly for light, asymmetric competition causes plant species intercepting more light early in the season to have a disproportionate advantage, leading to competitive exclusion of subordinate species (DeMalach et al., 2017; Hautier, 2009; Hautier, Vojtech, et al., 2018; Vojtech et al., 2007, 2008). Our study is the first to our knowledge to reveal the critical time during the growing season when exclusion mechanisms act. We show that difference in early‐season growth rates provides an explanation of competitive outcomes, thereby serving as a predictor and early signaling of plant competitive abilities. This is because under productive conditions, asymmetric competition leads to increased relative size differences between species early in the season. This early advantage allows fast‐growing species to maintain and increase their initial dominant position throughout the growing season, leading to the exclusion of initially slower‐growing species. Our study is in agreement with earlier studies demonstrating that instantaneous measurements of light obtained early in the season, at the critical time when light becomes limiting for plant growth, were the best predictors of competitive outcomes (Violle, Lecoeur, & Navas, 2007; Vojtech et al., 2007).

Our results from the field experiment are based on a subset of the total number of species occurring in the community. Growth rates were derived from the twenty most common species across all treatments, accounting for 85 ± 10% of the total aboveground biomass. Our results are therefore most likely conservative because they are restricted to competitive exclusion among the twenty most common species, thereby failing to consider the exclusion of the rarest species, which comprise a large proportion of the total species number and are more susceptible to human disturbances (Zhang et al., 2019).

Previous studies have shown that the outcome of competition in pairwise mixtures could be best predicted by differences in light intercepting ability in monocultures (I*) under productive (light‐limited) conditions and by differences in nutrient uptake ability in monocultures (R*) under unproductive conditions (Dybzinski & Tilman, 2007; Hautier, Vojtech, et al., 2018; Vojtech et al., 2007). However, in real‐world ecosystems that encompass nutrient gradients, both forms of competition are likely to act at the same time, with light competition becoming more important as nutrient competition lessens. Our results are consistent with the resource ratio hypothesis envisaging a trade‐off between competition for light under fertile conditions and for nutrients under less fertile conditions. Under fertile conditions, species growing faster early in the season have a competitive advantage over initially slower‐growing species (consistent with them being better competitors for light). This relationship between RGR and competitive success weakens under less fertile conditions (compare fertile conditions with added nitrogen from less fertile conditions without added nitrogen in Figures 1, 2 S3, S5, and S6). However, we would expect, based on earlier work (Tilman & Wedin, 1991; Wedin & Tilman, 1993), that slow‐growing species with the lowest R* for soil resources would dominate the community in the long term (a long‐term outcome that we were not able to assess in our relatively short‐term study). This would require that slow‐growing species do not entirely disappear from the landscape.

Our study thus suggests that human activities that increase the availability of nutrients to ecosystems will likely further reduce plant diversity in the future by benefitting initially fast‐growing species. In contrast, management practices directed toward reducing soil fertility in productive ecosystems could reduce the growth of fast‐growing species early in the season and help efforts to protect and restore biodiversity in an increasingly human‐dominated world. For example, repeated mowing and removing of the harvested plant material can help removing excess accumulated nutrients in the soils, allowing the subsequent recovery of diversity (Storkey et al., 2015). Alternatively, low‐diversity stable state could persist even after decades of cessation of nutrient enrichment if biomass is not removed and recycled within the system (Isbell, Tilman, Polasky, Binder, & Hawthorne, 2013; Tilman & Isbell, 2015). Removing the topsoil, typically between 20 and 50 cm (Frouz et al., 2009), can also help converting intensively managed grasslands into species‐rich grasslands, especially if combined with introduction of propagules of target plant species (Kiehl, Kirmer, Donath, Rasran, & Holzel, 2010; Kiehl & Pfadenhauer, 2007). Additionally, parasitic plants such as *Rhinanthus* species can restore biodiversity in productive grasslands (Bardgett et al., 2006; Bullock & Pywell, 2005; DiGiovanni, Wysocki, Burke, Duvall, & Barber, 2017; Pywell et al., 2004). They represent a practical restoration tool due to the low cost and accessibility of seeds, the rapid population growth and spread, and the possibility to limit the population size as needed (Bullock & Pywell, 2005). A potential mechanism is through the reduction of the biomass of competitively dominant grasses (Ameloot, Verheyen, & Hermy, 2005; Davies, Graves, Elias, & Williams, 1997), simply because the parasite reduces host resources leading to a reduction in host growth rate and future resource uptake (Hautier, Hector, Vojtech, Purves, & Turnbull, 2010). Our results suggest that *Rhinanthus* species could be particularly effective because they cancel out the initial advantage of fast‐growing species early in the season thus limiting the exclusion of slower‐growing species.

## CONFLICT OF INTEREST

None declared.

## AUTHOR CONTRIBUTIONS

**Pengfei Zhang:** Conceptualization (lead); formal analysis (lead); investigation (lead); methodology (lead); writing – original draft (lead); writing – review and editing (lead). **Mariet M. Hefting:** Conceptualization (supporting); formal analysis (supporting); supervision (supporting); writing – original draft (supporting); writing – review and editing (supporting). **Merel B. Soons:** Conceptualization (supporting); formal analysis (supporting); writing – original draft (supporting); writing – review and editing (supporting). **George A. Kowalchuk:** Conceptualization (supporting); formal analysis (supporting); supervision (supporting); writing – original draft (supporting); writing – review and editing (supporting). **Mark Rees:** Conceptualization (supporting); formal analysis (supporting); writing – original draft (supporting); writing – review and editing (supporting). **Andy Hector:** Conceptualization (supporting); formal analysis (supporting); resources (lead); writing – original draft (supporting); writing – review and editing (supporting). **Lindsay A. Turnbull:** Conceptualization (supporting); formal analysis (supporting); writing – original draft (supporting); writing – review and editing (supporting). **Xiaolong Zhou:** Investigation (equal); methodology (equal); writing – original draft (supporting); writing – review and editing (supporting). **Zhi Guo:** Investigation (equal); methodology (equal); writing – original draft (supporting); writing – review and editing (supporting). **Chengjing Chu:** Investigation (equal); methodology (equal); writing – original draft (supporting); writing – review and editing (supporting). **Guozhen Du:** Funding acquisition (lead); project administration (lead); resources (lead); supervision (equal); writing – original draft (supporting); writing – review and editing (supporting). **Yann Hautier:** Conceptualization (lead); data curation (equal); formal analysis (equal); investigation (equal); methodology (equal); project administration (lead); supervision (lead); writing – original draft (lead); writing – review and editing (lead).

### Open Research Badges

This article has earned an Open Data Badge for making publicly available the digitally‐shareable data necessary to reproduce the reported results. The data is available at https://doi.org/10.5061/dryad.95x69p8h9 and https://doi.org/10.5061/dryad.djh9w0vxm.

## Supporting information

Appendix S1

## Data Availability

Data from the common garden experiment of aboveground plant biomass collected during sequential harvests on days 53, 67, 88, 109, 116, 123, 130, 145, 152, 162, and 171 in the year 2008 are available at: https://doi.org/10.5061/dryad.95x69p8h9. Data from the field experiment of aboveground plant biomass collected during sequential harvests on days 146, 157, 167, 177, 197, 207, 238, and 254 in the year of 2013 are available at: https://doi.org/10.5061/dryad.djh9w0vxm.
