# Supplementary material for: Fast and furious: Early differences in growth rate drive short‐term plant dominance and exclusion under eutrophication
Source: Ecol Evol. 2020 Sep 9;10(18):10116–29. doi: 10.1002/ece3.6673 (PMC7520198; doi:10.1002/ece3.6673)
Supplement: Supplementary file 1 — Appendix S1 [file ECE3-10-10116-s001.docx]

**Appendix S1**

Fast and furious: Early differences in growth rate drive short-term plant dominance and exclusion under eutrophication

Pengfei Zhang, Mariet M. Hefting, Merel B. Soons, George A. Kowalchuk, Mark Rees, Andy Hector, Lindsay A. Turnbull, Xiaolong Zhou, Zhi Guo, Chengjing Chu, Guozhen Du, Yann Hautier


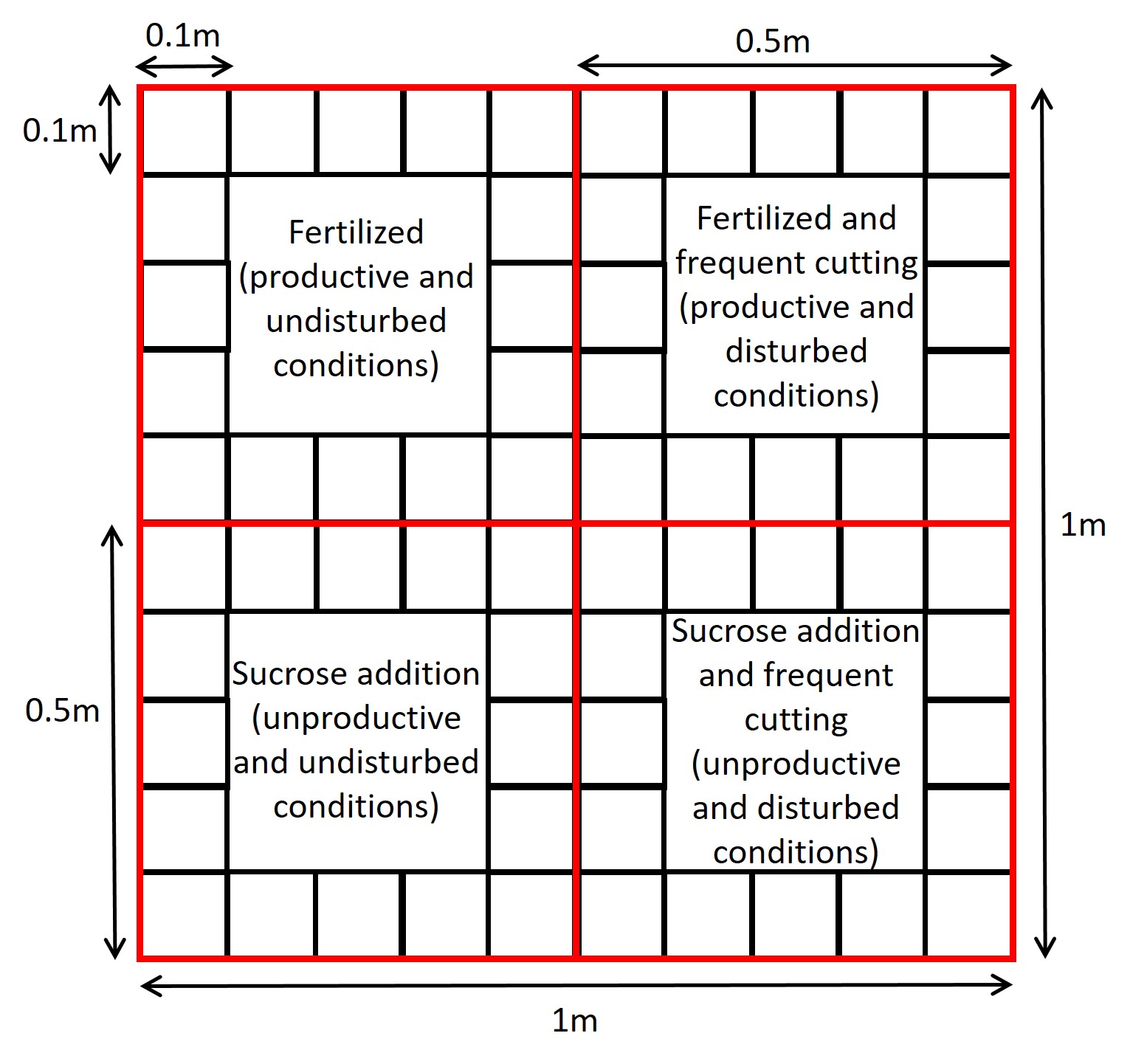


**Figure S1.** **Common garden.** In 2007, plots of 1m^2^ were divided into four subplots of 50 x 50 cm. We applied two treatments in a fully-factorial design: addition of sucrose and frequent cutting of the canopy structure. The subplots that did not receive sucrose were continuously fertilized with an NPK fertilizer. This design led to four treatments varying in their productivity and disturbance conditions: Productive undisturbed, unproductive undisturbed, productive disturbed, and productive undisturbed. Aboveground plant biomass at harvest was measured in the inner 30 x 30 cm of each subplot. Daily RGR of each species in monoculture was calculated based on aboveground plant biomass measured within 10 x 10 cm quadrats in the outer 10 cm surrounding the inner 30 x 30 cm of each subplot during sequential harvests. Each time different randomly chosen quadrats were measured. Calculating daily RGR per species throughout the growing season for the plots that were disturbed was not possible because of the limited number of samples between each cutting event. Here, we therefore analyse only the undisturbed productive and unproductive conditions.


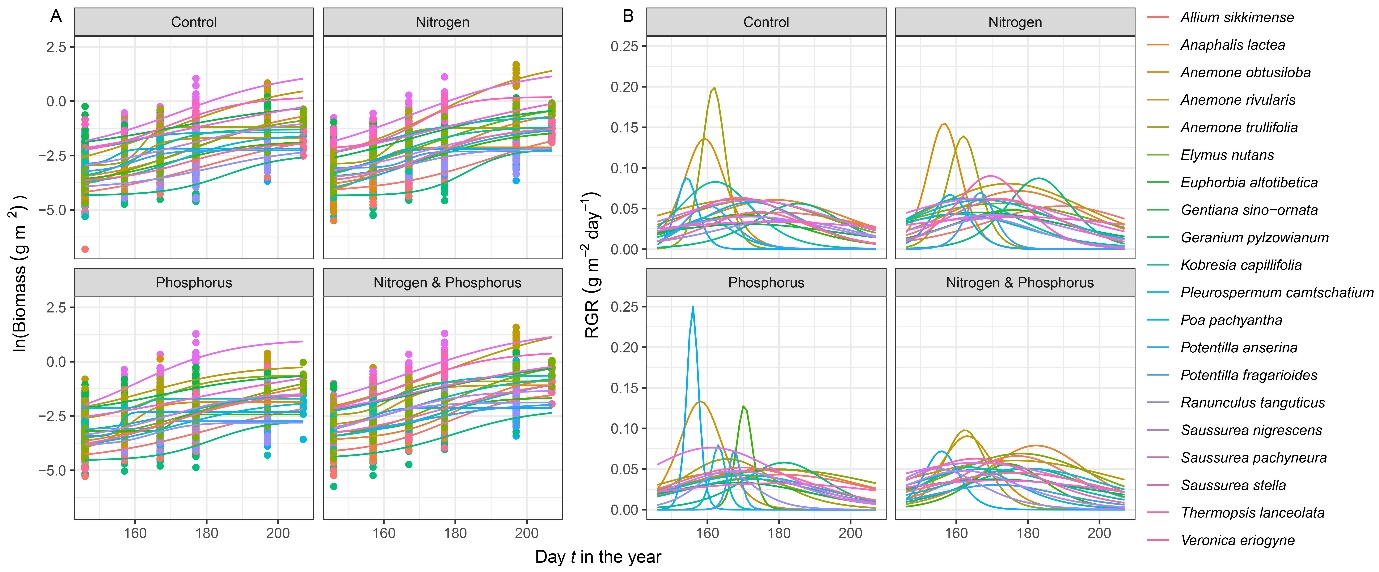


**Figure S2.** **Field experiment.** Fitted curves predicted from a four-parameter logistic model for A) biomass (log transformed) and B) relative growth rate (RGR) over time for twenty common species in a field experiment which combined addition of nitrogen and phosphorus in a full factorial design. Within each graph (A, B) fertile conditions with added N (right) are separated from less fertile conditions without added N (left).

**
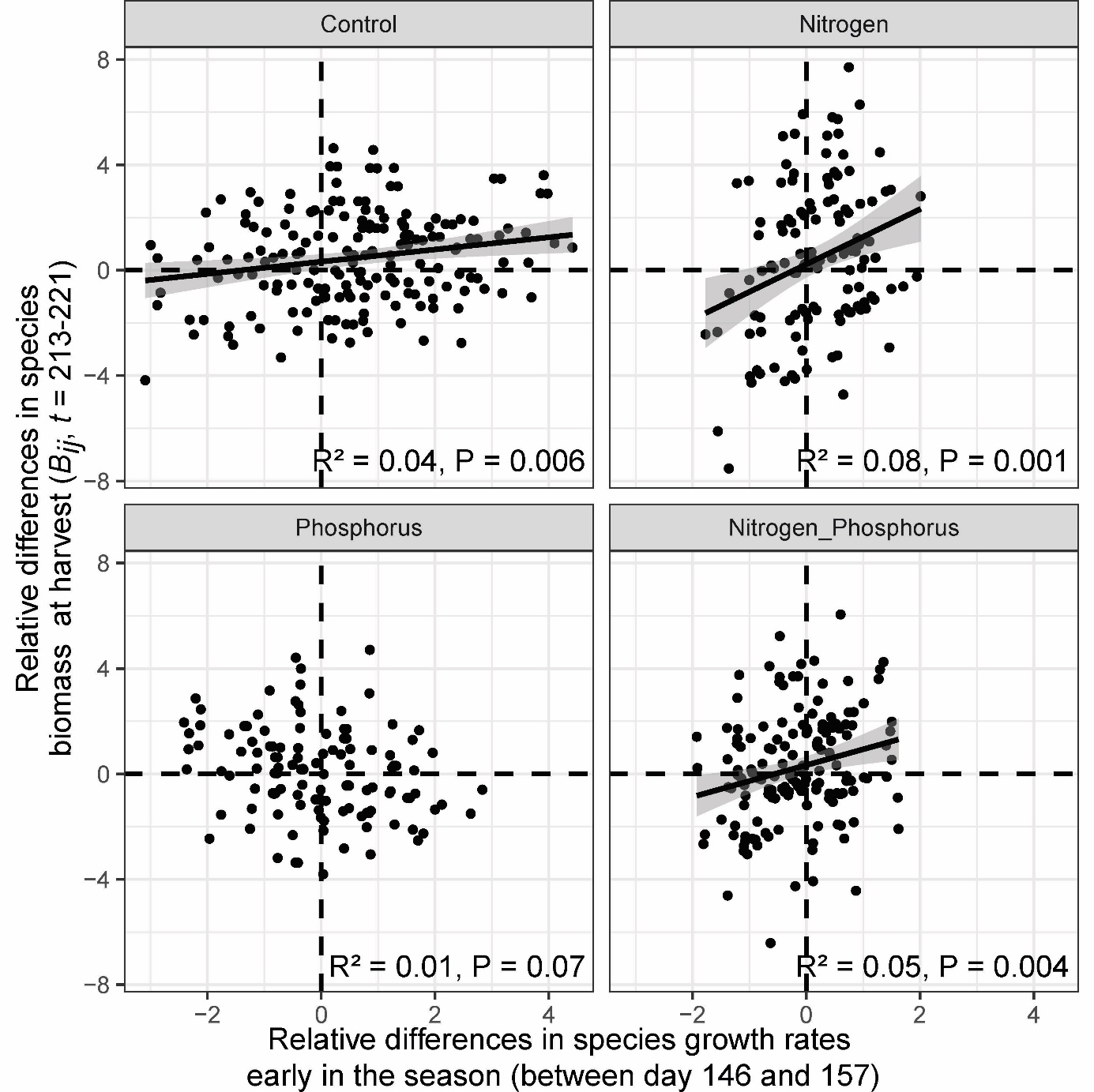
Figure S3.** **Field experiment.** RGR calculated between the two first measurements of biomass predicts competitive dominance. Early-season relative differences in species growth rates (measured between day 146 and 157) in a nutrient addition combination predict relative differences in species biomass in pairs of species combinations of the respective nutrient addition combination ($B_{ij}$) at harvest date ($t=213-221$) except in the phosphorus treatment. Fertile conditions with added N (right) are separated from less fertile conditions without added N (left). The grey region indicates the 95% confidence interval around the regression.

**Table S1.** **Field experiment.** The rank of the 20 common species (based on the percentage of total biomass of each species across all treatments) sampled.

| **Rank** | **Species** | **Functional group** | **Percentage of total biomass (mean ± SD)** | **Cumulative percentage of total biomass** |
| --- | --- | --- | --- | --- |
| 1 | *Kobresia capillifolia* | Sedges | 28.5% ± 15.6% | 29% |
| 2 | *Anemone rivularis* | Forbs | 20.3% ± 18.6% | 49% |
| 3 | *Elymus nutans* | Grasses | 13.0% ± 7.6% | 62% |
| 4 | *Pleurospermum camtschatium* | Forbs | 5.8% ± 5.6% | 68% |
| 5 | *Saussurea stella* | Forbs | 3.5% ± 3.3% | 71% |
| 6 | *Saussurea nigrescens* | Forbs | 3.3% ± 2.1% | 75% |
| 7 | *Anaphalis lactea* | Forbs | 1.9% ± 1.6% | 77% |
| 8 | *Thermopsis lanceolata* | Legumes | 1.4% ± 1.7% | 78% |
| 9 | *Potentilla fragarioides* | Forbs | 1.1% ± 0.8% | 79% |
| 10 | *Gentiana sino-ornata* | Forbs | 0.8% ± 0.7% | 80% |
| 11 | *Anemone obtusiloba* | Forbs | 0.8% ± 1.6% | 81% |
| 12 | *Allium sikkimense* | Forbs | 0.7% ± 2.0% | 81% |
| 13 | *Anemone trullifolia* | Forbs | 0.7% ± 1.5% | 82% |
| 14 | *Euphorbia altotibetica* | Forbs | 0.6% ± 0.7% | 83% |
| 15 | *Potentilla anserina* | Forbs | 0.5% ± 0.7% | 83% |
| 16 | *Saussurea pachyneura* | Forbs | 0.5% ± 1.1% | 84% |
| 17 | *Poa pachyantha* | Grasses | 0.5% ± 1.1% | 84% |
| 18 | *Geranium pylzowianum* | Forbs | 0.3% ± 0.6% | 85% |
| 19 | *Veronica eriogyne* | Forbs | 0.2% ± 0.4% | 85% |
| 20 | *Ranunculus tanguticus* | Forbs | 0.1% ± 0.3% | 85% |

**Table S2.** **Common garden.** Linear model estimates of the effect of two years of NPK fertilizer (NPK) and sucrose addition (Sucrose) on aboveground plant biomass at harvest (Biomass), light transmitted at the soil surface before the harvest (Light), and mineral nitrogen (nitrate + ammonium) availability before the harvest (Nmin) averaged over five grass monocultures in 2008.

| Biomass (g m^-2^) |  |  |  |  |
| --- | --- | --- | --- | --- |
|  | Estimate | Std. Error | t-value | p-value |
| NPK (Intercept) | 744.97 | 38.87 | 19.16 | <0.0001 |
| Sucrose | -470.63 | 46.52 | -10.12 | <0.0001 |
|  |  |  |  |  |
| Light (%) – (logit scale) |  |  |  |  |
|  | Estimate | Std. Error | t-value | p-value |
| NPK (Intercept) | -1.92 | 0.32 | -5.99 | <0.0001 |
| Sucrose | 2.56 | 0.39 | 6.53 | <0.0001 |
|  |  |  |  |  |
| Nmin (g m^-2^) |  |  |  |  |
|  | Estimate | Std. Error | t-value | p-value |
| NPK fertilizer (Intercept) | 2.20 | 0.49 | 4.51 | 0.002 |
| Sucrose | -1.48 | 0.53 | -2.78 | 0.02 |

**Table S3.** **Common garden.** Nonlinear mixed-effect model estimates of the four-parameter logistic growth fitted to biomass data through time with species, productivity treatment and their interaction as random effect.

|  | Estimate | Std. Error | t-value | p-value |
| --- | --- | --- | --- | --- |
| $M_{0}$ (Intercept) | 6.95 | 0.12 | 60.29 | <0.01 |
| $M_{0}$ speciesAn | -0.05 | 0.16 | -0.33 | 0.74 |
| $M_{0}$ speciesAr | -0.85 | 0.18 | -4.84 | <0.01 |
| $M_{0}$ speciesF | -0.79 | 0.16 | -5.06 | <0.01 |
| $M_{0}$ speciesH | -0.11 | 0.18 | -0.60 | 0.55 |
| $M_{0}$ trtSucrose | -0.02 | 0.15 | -0.12 | 0.90 |
| $M_{0}$ speciesAn:trtSucrose | 0.00 | 0.19 | -0.02 | 0.98 |
| $M_{0}$ speciesAr:trtSucrose | -0.10 | 0.23 | -0.43 | 0.67 |
| $M_{0}$ speciesF:trtSucrose | 0.27 | 0.18 | 1.47 | 0.14 |
| $M_{0}$ speciesH:trtSucrose | -0.31 | 0.23 | -1.36 | 0.18 |
| $K$ (Intercept) | 9.24 | 0.14 | 66.01 | <0.01 |
| $K$ speciesAn | -0.18 | 0.23 | -0.79 | 0.43 |
| $K$ speciesAr | -0.47 | 0.20 | -2.35 | 0.02 |
| $K$ speciesF | -0.21 | 0.19 | -1.08 | 0.28 |
| $K$ speciesH | 1.13 | 0.48 | 2.37 | 0.02 |
| $K$ trtSucrose | -0.10 | 0.14 | -0.72 | 0.47 |
| $K$ speciesAn:trtSucrose | -0.08 | 0.25 | -0.33 | 0.74 |
| $K$ speciesAr:trtSucrose | -0.35 | 0.30 | -1.18 | 0.24 |
| $K$ speciesF:trtSucrose | -0.43 | 0.22 | -1.93 | 0.05 |
| $K$ speciesH:trtSucrose | -1.11 | 0.50 | -2.21 | 0.03 |
| $xmid$ (Intercept) | 134.35 | 4.05 | 33.17 | <0.01 |
| $xmid$ speciesAn | 9.85 | 5.80 | 1.70 | 0.09 |
| $xmid$ speciesAr | 0.14 | 5.68 | 0.02 | 0.98 |
| $xmid$ speciesF | 12.73 | 5.38 | 2.37 | 0.02 |
| $xmid$ speciesH | 16.62 | 7.61 | 2.18 | 0.03 |
| $xmid$ trtSucrose | -9.68 | 3.24 | -2.99 | <0.01 |
| $xmid$ speciesAn:trtSucrose | 8.58 | 4.48 | 1.91 | 0.06 |
| $xmid$ speciesAr:trtSucrose | 15.97 | 5.49 | 2.91 | <0.01 |
| $xmid$ speciesF:trtSucrose | 11.24 | 3.74 | 3.01 | <0.01 |
| $xmid$ speciesH:trtSucrose | -2.21 | 7.20 | -0.31 | 0.76 |
| $r$ (Intercept) | 11.83 | 2.20 | 5.37 | <0.01 |
| $r$ speciesAn | -1.92 | 3.20 | -0.60 | 0.55 |
| $r$ speciesAr | -1.29 | 2.92 | -0.44 | 0.66 |
| $r$ speciesF | -5.86 | 2.55 | -2.30 | 0.02 |
| $r$ speciesH | 8.54 | 4.48 | 1.91 | 0.06 |
| $r$ trtSucrose | 1.13 | 3.17 | 0.36 | 0.72 |
| $r$ speciesAn:trtSucrose | -2.82 | 4.37 | -0.64 | 0.52 |
| $r$ speciesAr:trtSucrose | 1.58 | 5.01 | 0.31 | 0.75 |
| $r$ speciesF:trtSucrose | -1.15 | 3.81 | -0.30 | 0.76 |
| $r$ speciesH:trtSucrose | -4.64 | 6.06 | -0.77 | 0.44 |

**Table S4.** **Common garden.** Results of the ANCOVA assessing whether early differences in growth rate between species in monocultures predict short-term competitive dominance at harvest (RGRt) in pairwise mixtures between day 53 and 171 in the season and whether this relationship changes under between productive and unproductive conditions (RGRt:trt).

| time | Df | F values RGRt | P values RGRt | F values trt | P values trt | F values RGRt:trt | P values RGRt:trt |
| --- | --- | --- | --- | --- | --- | --- | --- |
| 53 | 1 | 89.74 | <0.01 | 1.59 | 0.21 | 0.08 | 0.77 |
| 54 | 1 | 89.89 | <0.01 | 1.58 | 0.21 | 0.08 | 0.78 |
| 55 | 1 | 90.05 | <0.01 | 1.58 | 0.21 | 0.08 | 0.78 |
| 56 | 1 | 90.21 | <0.01 | 1.58 | 0.21 | 0.08 | 0.78 |
| 57 | 1 | 90.37 | <0.01 | 1.58 | 0.21 | 0.08 | 0.78 |
| 58 | 1 | 90.54 | <0.01 | 1.58 | 0.21 | 0.08 | 0.78 |
| 59 | 1 | 90.71 | <0.01 | 1.57 | 0.21 | 0.08 | 0.78 |
| 60 | 1 | 90.88 | <0.01 | 1.57 | 0.21 | 0.07 | 0.79 |
| 61 | 1 | 91.05 | <0.01 | 1.57 | 0.21 | 0.07 | 0.79 |
| 62 | 1 | 91.23 | <0.01 | 1.57 | 0.21 | 0.07 | 0.79 |
| 63 | 1 | 91.41 | <0.01 | 1.56 | 0.21 | 0.07 | 0.79 |
| 64 | 1 | 91.59 | <0.01 | 1.56 | 0.21 | 0.07 | 0.79 |
| 65 | 1 | 91.77 | <0.01 | 1.56 | 0.21 | 0.07 | 0.8 |
| 66 | 1 | 91.96 | <0.01 | 1.56 | 0.22 | 0.07 | 0.8 |
| 67 | 1 | 92.14 | <0.01 | 1.55 | 0.22 | 0.06 | 0.8 |
| 68 | 1 | 92.33 | <0.01 | 1.55 | 0.22 | 0.06 | 0.8 |
| 69 | 1 | 92.53 | <0.01 | 1.55 | 0.22 | 0.06 | 0.81 |
| 70 | 1 | 92.72 | <0.01 | 1.55 | 0.22 | 0.06 | 0.81 |
| 71 | 1 | 92.92 | <0.01 | 1.54 | 0.22 | 0.06 | 0.81 |
| 72 | 1 | 93.12 | <0.01 | 1.54 | 0.22 | 0.06 | 0.81 |
| 73 | 1 | 93.32 | <0.01 | 1.54 | 0.22 | 0.05 | 0.82 |
| 74 | 1 | 93.52 | <0.01 | 1.53 | 0.22 | 0.05 | 0.82 |
| 75 | 1 | 93.72 | <0.01 | 1.53 | 0.22 | 0.05 | 0.82 |
| 76 | 1 | 93.93 | <0.01 | 1.53 | 0.22 | 0.05 | 0.82 |
| 77 | 1 | 94.13 | <0.01 | 1.53 | 0.22 | 0.05 | 0.83 |
| 78 | 1 | 94.34 | <0.01 | 1.52 | 0.22 | 0.05 | 0.83 |
| 79 | 1 | 94.54 | <0.01 | 1.52 | 0.22 | 0.05 | 0.83 |
| 80 | 1 | 94.75 | <0.01 | 1.52 | 0.22 | 0.04 | 0.83 |
| 81 | 1 | 94.95 | <0.01 | 1.51 | 0.22 | 0.04 | 0.84 |
| 82 | 1 | 95.15 | <0.01 | 1.51 | 0.22 | 0.04 | 0.84 |
| 83 | 1 | 95.35 | <0.01 | 1.51 | 0.22 | 0.04 | 0.84 |
| 84 | 1 | 95.55 | <0.01 | 1.51 | 0.22 | 0.04 | 0.85 |
| 85 | 1 | 95.74 | <0.01 | 1.5 | 0.22 | 0.04 | 0.85 |
| 86 | 1 | 95.93 | <0.01 | 1.5 | 0.22 | 0.03 | 0.85 |
| 87 | 1 | 96.12 | <0.01 | 1.5 | 0.22 | 0.03 | 0.86 |
| 88 | 1 | 96.3 | <0.01 | 1.49 | 0.22 | 0.03 | 0.86 |
| 89 | 1 | 96.47 | <0.01 | 1.49 | 0.23 | 0.03 | 0.86 |
| 90 | 1 | 96.63 | <0.01 | 1.49 | 0.23 | 0.03 | 0.86 |
| 91 | 1 | 96.78 | <0.01 | 1.49 | 0.23 | 0.03 | 0.87 |
| 92 | 1 | 96.92 | <0.01 | 1.48 | 0.23 | 0.03 | 0.87 |
| 93 | 1 | 97.05 | <0.01 | 1.48 | 0.23 | 0.03 | 0.87 |
| 94 | 1 | 97.17 | <0.01 | 1.48 | 0.23 | 0.02 | 0.88 |
| 95 | 1 | 97.26 | <0.01 | 1.48 | 0.23 | 0.02 | 0.88 |
| 96 | 1 | 97.34 | <0.01 | 1.48 | 0.23 | 0.02 | 0.88 |
| 97 | 1 | 97.39 | <0.01 | 1.47 | 0.23 | 0.02 | 0.89 |
| 98 | 1 | 97.42 | <0.01 | 1.47 | 0.23 | 0.02 | 0.89 |
| 99 | 1 | 97.43 | <0.01 | 1.47 | 0.23 | 0.02 | 0.89 |
| 100 | 1 | 97.4 | <0.01 | 1.47 | 0.23 | 0.02 | 0.89 |
| 101 | 1 | 97.34 | <0.01 | 1.47 | 0.23 | 0.02 | 0.9 |
| 102 | 1 | 97.24 | <0.01 | 1.47 | 0.23 | 0.02 | 0.9 |
| 103 | 1 | 97.1 | <0.01 | 1.47 | 0.23 | 0.02 | 0.9 |
| 104 | 1 | 96.91 | <0.01 | 1.47 | 0.23 | 0.02 | 0.9 |
| 105 | 1 | 96.67 | <0.01 | 1.47 | 0.23 | 0.01 | 0.9 |
| 106 | 1 | 96.38 | <0.01 | 1.47 | 0.23 | 0.01 | 0.9 |
| 107 | 1 | 96.02 | <0.01 | 1.47 | 0.23 | 0.01 | 0.91 |
| 108 | 1 | 95.59 | <0.01 | 1.47 | 0.23 | 0.01 | 0.91 |
| 109 | 1 | 95.08 | <0.01 | 1.47 | 0.23 | 0.01 | 0.91 |
| 110 | 1 | 94.49 | <0.01 | 1.47 | 0.23 | 0.01 | 0.91 |
| 111 | 1 | 93.81 | <0.01 | 1.47 | 0.23 | 0.01 | 0.91 |
| 112 | 1 | 93.02 | <0.01 | 1.48 | 0.23 | 0.01 | 0.91 |
| 113 | 1 | 92.13 | <0.01 | 1.48 | 0.23 | 0.01 | 0.91 |
| 114 | 1 | 91.1 | <0.01 | 1.48 | 0.23 | 0.01 | 0.9 |
| 115 | 1 | 89.94 | <0.01 | 1.48 | 0.23 | 0.01 | 0.9 |
| 116 | 1 | 88.62 | <0.01 | 1.48 | 0.23 | 0.02 | 0.9 |
| 117 | 1 | 87.14 | <0.01 | 1.48 | 0.23 | 0.02 | 0.9 |
| 118 | 1 | 85.46 | <0.01 | 1.48 | 0.23 | 0.02 | 0.89 |
| 119 | 1 | 83.56 | <0.01 | 1.48 | 0.23 | 0.02 | 0.89 |
| 120 | 1 | 81.43 | <0.01 | 1.48 | 0.23 | 0.02 | 0.89 |
| 121 | 1 | 79.02 | <0.01 | 1.48 | 0.23 | 0.02 | 0.88 |
| 122 | 1 | 76.29 | <0.01 | 1.48 | 0.23 | 0.03 | 0.87 |
| 123 | 1 | 73.21 | <0.01 | 1.47 | 0.23 | 0.03 | 0.87 |
| 124 | 1 | 69.7 | <0.01 | 1.46 | 0.23 | 0.03 | 0.86 |
| 125 | 1 | 65.71 | <0.01 | 1.45 | 0.23 | 0.04 | 0.84 |
| 126 | 1 | 61.15 | <0.01 | 1.44 | 0.23 | 0.05 | 0.82 |
| 127 | 1 | 55.92 | <0.01 | 1.42 | 0.24 | 0.07 | 0.79 |
| 128 | 1 | 49.91 | <0.01 | 1.39 | 0.24 | 0.1 | 0.76 |
| 129 | 1 | 43.01 | <0.01 | 1.36 | 0.25 | 0.15 | 0.7 |
| 130 | 1 | 35.15 | <0.01 | 1.33 | 0.25 | 0.25 | 0.62 |
| 131 | 1 | 26.35 | <0.01 | 1.3 | 0.26 | 0.44 | 0.51 |
| 132 | 1 | 16.92 | <0.01 | 1.27 | 0.26 | 0.79 | 0.38 |
| 133 | 1 | 7.81 | <0.01 | 1.25 | 0.27 | 1.31 | 0.25 |

**Table S5.** **Common garden.** Results of the ANCOVA assessing whether early differences in growth rate between species in monocultures predict short-term competitive dominance at harvest (RGRt) in five-species mixtures between day 53 and 171 in the season and whether this relationship changes under between productive and unproductive conditions (RGRt:trt).

| time | Df | F values RGRt | P values RGRt | F values trt | P values trt | F values RGRt:trt | P values RGRt:trt |
| --- | --- | --- | --- | --- | --- | --- | --- |
| 53 | 1 | 104.41 | <0.01 | 21.62 | <0.01 | 3.47 | 0.07 |
| 54 | 1 | 104.7 | <0.01 | 21.64 | <0.01 | 3.5 | 0.06 |
| 55 | 1 | 105.01 | <0.01 | 21.65 | <0.01 | 3.53 | 0.06 |
| 56 | 1 | 105.31 | <0.01 | 21.67 | <0.01 | 3.56 | 0.06 |
| 57 | 1 | 105.63 | <0.01 | 21.69 | <0.01 | 3.59 | 0.06 |
| 58 | 1 | 105.95 | <0.01 | 21.71 | <0.01 | 3.62 | 0.06 |
| 59 | 1 | 106.28 | <0.01 | 21.72 | <0.01 | 3.65 | 0.06 |
| 60 | 1 | 106.62 | <0.01 | 21.74 | <0.01 | 3.68 | 0.06 |
| 61 | 1 | 106.97 | <0.01 | 21.76 | <0.01 | 3.71 | 0.06 |
| 62 | 1 | 107.32 | <0.01 | 21.78 | <0.01 | 3.75 | 0.06 |
| 63 | 1 | 107.68 | <0.01 | 21.8 | <0.01 | 3.78 | 0.05 |
| 64 | 1 | 108.05 | <0.01 | 21.83 | <0.01 | 3.82 | 0.05 |
| 65 | 1 | 108.42 | <0.01 | 21.85 | <0.01 | 3.86 | 0.05 |
| 66 | 1 | 108.81 | <0.01 | 21.87 | <0.01 | 3.89 | 0.05 |
| 67 | 1 | 109.2 | <0.01 | 21.89 | <0.01 | 3.93 | 0.05 |
| 68 | 1 | 109.61 | <0.01 | 21.92 | <0.01 | 3.97 | 0.05 |
| 69 | 1 | 110.02 | <0.01 | 21.94 | <0.01 | 4.02 | 0.05 |
| 70 | 1 | 110.44 | <0.01 | 21.97 | <0.01 | 4.06 | 0.05 |
| 71 | 1 | 110.87 | <0.01 | 21.99 | <0.01 | 4.1 | 0.05 |
| 72 | 1 | 111.31 | <0.01 | 22.02 | <0.01 | 4.15 | 0.04 |
| 73 | 1 | 111.76 | <0.01 | 22.05 | <0.01 | 4.19 | 0.04 |
| 74 | 1 | 112.22 | <0.01 | 22.07 | <0.01 | 4.24 | 0.04 |
| 75 | 1 | 112.69 | <0.01 | 22.1 | <0.01 | 4.29 | 0.04 |
| 76 | 1 | 113.17 | <0.01 | 22.13 | <0.01 | 4.34 | 0.04 |
| 77 | 1 | 113.66 | <0.01 | 22.17 | <0.01 | 4.4 | 0.04 |
| 78 | 1 | 114.16 | <0.01 | 22.2 | <0.01 | 4.45 | 0.04 |
| 79 | 1 | 114.67 | <0.01 | 22.23 | <0.01 | 4.51 | 0.04 |
| 80 | 1 | 115.19 | <0.01 | 22.26 | <0.01 | 4.56 | 0.04 |
| 81 | 1 | 115.72 | <0.01 | 22.3 | <0.01 | 4.62 | 0.03 |
| 82 | 1 | 116.26 | <0.01 | 22.34 | <0.01 | 4.68 | 0.03 |
| 83 | 1 | 116.8 | <0.01 | 22.37 | <0.01 | 4.75 | 0.03 |
| 84 | 1 | 117.36 | <0.01 | 22.41 | <0.01 | 4.81 | 0.03 |
| 85 | 1 | 117.93 | <0.01 | 22.45 | <0.01 | 4.88 | 0.03 |
| 86 | 1 | 118.51 | <0.01 | 22.49 | <0.01 | 4.95 | 0.03 |
| 87 | 1 | 119.09 | <0.01 | 22.54 | <0.01 | 5.02 | 0.03 |
| 88 | 1 | 119.69 | <0.01 | 22.58 | <0.01 | 5.09 | 0.03 |
| 89 | 1 | 120.29 | <0.01 | 22.63 | <0.01 | 5.17 | 0.03 |
| 90 | 1 | 120.89 | <0.01 | 22.67 | <0.01 | 5.24 | 0.02 |
| 91 | 1 | 121.5 | <0.01 | 22.72 | <0.01 | 5.32 | 0.02 |
| 92 | 1 | 122.11 | <0.01 | 22.77 | <0.01 | 5.4 | 0.02 |
| 93 | 1 | 122.73 | <0.01 | 22.83 | <0.01 | 5.49 | 0.02 |
| 94 | 1 | 123.34 | <0.01 | 22.88 | <0.01 | 5.57 | 0.02 |
| 95 | 1 | 123.96 | <0.01 | 22.93 | <0.01 | 5.66 | 0.02 |
| 96 | 1 | 124.57 | <0.01 | 22.99 | <0.01 | 5.75 | 0.02 |
| 97 | 1 | 125.17 | <0.01 | 23.05 | <0.01 | 5.84 | 0.02 |
| 98 | 1 | 125.76 | <0.01 | 23.11 | <0.01 | 5.94 | 0.02 |
| 99 | 1 | 126.35 | <0.01 | 23.17 | <0.01 | 6.03 | 0.02 |
| 100 | 1 | 126.91 | <0.01 | 23.23 | <0.01 | 6.13 | 0.02 |
| 101 | 1 | 127.45 | <0.01 | 23.3 | <0.01 | 6.23 | 0.01 |
| 102 | 1 | 127.97 | <0.01 | 23.36 | <0.01 | 6.33 | 0.01 |
| 103 | 1 | 128.45 | <0.01 | 23.43 | <0.01 | 6.43 | 0.01 |
| 104 | 1 | 128.89 | <0.01 | 23.49 | <0.01 | 6.53 | 0.01 |
| 105 | 1 | 129.29 | <0.01 | 23.56 | <0.01 | 6.64 | 0.01 |
| 106 | 1 | 129.64 | <0.01 | 23.62 | <0.01 | 6.74 | 0.01 |
| 107 | 1 | 129.92 | <0.01 | 23.69 | <0.01 | 6.84 | 0.01 |
| 108 | 1 | 130.13 | <0.01 | 23.76 | <0.01 | 6.95 | 0.01 |
| 109 | 1 | 130.26 | <0.01 | 23.82 | <0.01 | 7.05 | 0.01 |
| 110 | 1 | 130.29 | <0.01 | 23.88 | <0.01 | 7.15 | 0.01 |
| 111 | 1 | 130.2 | <0.01 | 23.93 | <0.01 | 7.25 | 0.01 |
| 112 | 1 | 129.99 | <0.01 | 23.99 | <0.01 | 7.35 | 0.01 |
| 113 | 1 | 129.64 | <0.01 | 24.03 | <0.01 | 7.44 | 0.01 |
| 114 | 1 | 129.12 | <0.01 | 24.07 | <0.01 | 7.53 | 0.01 |
| 115 | 1 | 128.4 | <0.01 | 24.09 | <0.01 | 7.61 | 0.01 |
| 116 | 1 | 127.47 | <0.01 | 24.11 | <0.01 | 7.69 | 0.01 |
| 117 | 1 | 126.3 | <0.01 | 24.1 | <0.01 | 7.76 | 0.01 |
| 118 | 1 | 124.83 | <0.01 | 24.08 | <0.01 | 7.82 | 0.01 |
| 119 | 1 | 123.04 | <0.01 | 24.04 | <0.01 | 7.86 | 0.01 |
| 120 | 1 | 120.87 | <0.01 | 23.96 | <0.01 | 7.89 | 0.01 |
| 121 | 1 | 118.26 | <0.01 | 23.85 | <0.01 | 7.9 | 0.01 |
| 122 | 1 | 115.14 | <0.01 | 23.7 | <0.01 | 7.88 | 0.01 |
| 123 | 1 | 111.43 | <0.01 | 23.49 | <0.01 | 7.83 | 0.01 |
| 124 | 1 | 107.01 | <0.01 | 23.22 | <0.01 | 7.72 | 0.01 |
| 125 | 1 | 101.75 | <0.01 | 22.87 | <0.01 | 7.55 | 0.01 |
| 126 | 1 | 95.51 | <0.01 | 22.41 | <0.01 | 7.28 | 0.01 |
| 127 | 1 | 88.11 | <0.01 | 21.83 | <0.01 | 6.88 | 0.01 |
| 128 | 1 | 79.36 | <0.01 | 21.09 | <0.01 | 6.3 | 0.01 |
| 129 | 1 | 69.08 | <0.01 | 20.16 | <0.01 | 5.48 | 0.02 |
| 130 | 1 | 57.2 | <0.01 | 19.02 | <0.01 | 4.38 | 0.04 |
| 131 | 1 | 43.85 | <0.01 | 17.65 | <0.01 | 3 | 0.09 |
| 132 | 1 | 29.64 | <0.01 | 16.13 | <0.01 | 1.51 | 0.22 |
| 133 | 1 | 15.83 | <0.01 | 14.57 | <0.01 | 0.35 | 0.55 |

**Table S6.** **Field experiment.** Linear model estimates of the effect of three years of N, P, and NP addition on aboveground plant biomass at peak biomass (Biomass), and the number of plant species (Richness) in 2013.

| Biomass (g 0.25 m^-2^) |  |  |  |  |
| --- | --- | --- | --- | --- |
|  | Estimate | Std.Error | t-value | p-value |
| Control (Intercept) | 100.56 | 7.25 | 13.87 | <0.0001 |
| Nitrogen | 39.04 | 13.67 | 2.86 | 0.0098 |
| Phosphorus | 13.47 | 8.98 | 1.50 | 0.15 |
| Nitrogen & Phosphorus | 44.70 | 22.90 | 61.95 | 0.065 |
|  |  |  |  |  |
| Richness (species 0.25 m^-2^) |  |  |  |  |
|  | Estimate | Std.Error | t-value | p-value |
| Control (Intercept) | 35.50 | 1.61 | 22.09 | <0.0001 |
| Nitrogen | -13.17 | 2.74 | -4.81 | <0.0001 |
| Phosphorus | -0.67 | 1.85 | -0.36 | 0.72 |
| Nitrogen & Phosphorus | 6.50 | 3.38 | 1.92 | 0.069 |
